# ID 209 - Impacto Orçamentário da Incorporação de Ganciclovir e Valganciclovir no Tratamento de Retinite por Citomegalovírus em Pessoas Vivendo com HIV/Aids na Perspectiva do SUS

**DOI:** 10.5327/2237-9622.2025.v34s1.209

**Published:** 2025-11-25

**Authors:** Bárbara Rodrigues Alvernaz dos Santos, Alex Brunno Martins, Ludmila Gargano, Roberto Muniz, Marcus Borin, Isabela Freitas, Francisco Acurcio, Juliana Alvares-Teodoro, Augusto Guerra-Júnior

**Keywords:** HIV, citomegalovírus, ganciclovir, valganciclovir, impacto orçamentário, SUS

## Abstract

**Introdução:**

O citomegalovírus (CMV) é um herpesvírus humano do tipo 5 e sua forma mais comum de transmissão CMV é através do contato íntimo ou com secreções de pessoas contaminadas. A infecção por CMV pode se manifestar de maneira sistemática ou em órgãos específicos. Em pessoas vivendo com HIV/aids, o comprometimento causado pela doença no sistema imunológico intensifica os sintomas provocados pela infecção por CMV, levando a quadros de colite, retinite e outros. A retinite é a manifestação mais comum da infecção por CMV em pessoas vivendo com HIV/aids e pode evoluir à cegueira irreversível pela destruição da retina. Estima-se que aproximadamente 1,7% dos pacientes com estágios avançados da aids desenvolvam a retinite em decorrência do CMV. No Brasil, ganciclovir intravenoso e valganciclovir oral são os medicamentos indicados para o tratamento do CMV e não estão disponíveis pelo Sistema Único de Saúde (SUS). Este trabalho teve como objetivo avaliar o impacto orçamentário da incorporação de ganciclovir e valganciclovir no tratamento de retinite por CMV no SUS.

**Método:**

Foi realizada uma análise de impacto orçamentário na perspectiva do SUS e com horizonte temporal de 5 anos. Para a análise, foram considerados apenas os custos diretos de aquisição dos medicamentos conforme aquisições pelo banco de preços em saúde. A população incluída refere-se às pessoas vivendo com HIV/aids com contagem de células TCD4+ <50 células/µl que apresentarão retinite por CMV (1,7%). Foram projetados dois cenários de difusão: o primeiro considerou a incorporação dos dois medicamentos e que, a cada ano, metade dos pacientes elegíveis utilizariam ganciclovir intravenoso e a outra metade utilizaria valganciclovir oral; o segundo cenário considerou a incorporação dos dois medicamentos, em que o ganciclovir iniciaria o primeiro ano com 80% da participação no mercado e essa participação reduziria 20% nos anos seguintes, chegando a 0% no último ano. Nesse cenário, o valganciclovir oral substituiria progressivamente o ganciclovir intravenoso.

**Resultados:**

No primeiro cenário, a incorporação dos dois antivirais compartilhando, cada um, metade do mercado, 50% cada, resultou em um custo acumulado de R$ 8.539.626,69 para o ganciclovir intravenoso, e de R$ 11.966.164,25 para o valganciclovir. No segundo cenário, o ganciclovir acumularia um custo total de R$ 6.794.744,26 para o ganciclovir e R$ 14.411.183,27 para o valganciclovir.

**Conclusão:**

O tratamento da retinite representa grande relevância clínica em pessoas vivendo com HIV/aids. A incorporação de ganciclovir e/ou valganciclovir representa uma esperança para os pacientes com essa condição. O ganciclovir intravenoso deve ser administrado em nível ambulatorial ou hospitalar, entretanto a análise de impacto orçamentário não considerou os custos de internação, administração, acessibilidade geográfica e qualidade de vida, o que pode subestimar o impacto orçamentário com o ganciclovir intravenoso. O uso de valganciclovir apresenta vantagens sobre a adesão terapêutica e a qualidade de vida tendo em vista a possibilidade de administração pelo próprio paciente.

